# Association of *GSTM1* Polymorphism and Redox Potential with Idiopathic Male Infertility

**DOI:** 10.3390/jcm12216775

**Published:** 2023-10-26

**Authors:** Anastasios Potiris, Anastasia Voitse, Despoina Mavrogianni, Nikolaos Machairiotis, Eirini Drakaki, Myrto Papamentzelopoulou, Theodoros Karampitsakos, Athanasios Zikopoulos, Evangelini Evgeni, Peter Drakakis, Sofoklis Stavros

**Affiliations:** 1 Third Department of Obstetrics and Gynecology, University General Hospital “ATTIKON”, Medical School, National and Kapodistrian University of Athens, 124 62 Athens, Greece; apotiris@med.uoa.gr (A.P.); nikolaosmachairiotis@gmail.com (N.M.); theokarampitsakos@hotmail.com (T.K.); pdrakakis@med.uoa.gr (P.D.); sfstavrou@med.uoa.gr (S.S.); 2 First Department of Obstetrics and Gynecology, Alexandra Hospital, Medical School, National and Kapodistrian University of Athens, 115 28 Athens, Greece; voitanast@med.uoa.gr (A.V.); eirinidrak@med.uoa.gr (E.D.); mpntua@yahoo.gr (M.P.); 3 Department of Obstetrics and Gynecology, Royal Cornwall Hospital, Treliske TR1 3LQ, UK; thanzik92@gmail.com; 4 Cryogonia Cryopreservation Bank, 115 26 Athens, Greece; lina.evgeni@cryogonia.gr

**Keywords:** *GSTM1* polymorphism, idiopathic male infertility, redox potential, semen parameters, biomarker

## Abstract

Background: The aim of this case–control study is to investigate possible associations between *GSTM1* polymorphism and redox potential with sperm parameters. Methods: The study group consisted of sperm samples from 51 infertile men according to the WHO guidelines. The control group included 39 samples from men with normal seminal parameters. DNA was extracted and genotyped for the detection of the *GSTM1* polymorphism. An evaluation of the static redox potential (sORP) using the MiOXSYS^TM^ system was conducted. Results: The frequency of the *GSTM1*-null genotype was higher in infertile male individuals (60.78%) than in the controls (41.03%) and was associated with a 2.228-fold increased risk for male infertility. Fertile controls carrying the *GSTM1*-null genotype presented a lower percentage of typical sperm morphology and lower slow progressive motility. An excess of redox potential was observed in infertile males compared to fertile ones. In the control group higher sORP values had a positive correlation with immotility percentage and a negative correlation regarding total motility. In the study group sORP values had a negative correlation with total count, concentration, and slow progressive motility. Conclusions: The present study highlights that *GSTM1* polymorphism and redox potential affect both fertile and in fertile males. Moreover, redox potential levels could be used as an additional indicator along with the routine semen analysis for a comprehensive screening between infertile and fertile men.

## 1. Introduction

Despite advances in male reproductive health, idiopathic male infertility remains a challenging condition in diagnosis and treatment. Increasing data suggest that oxidative stress plays a major role in the pathophysiology of male infertility, with 30% to 80% of infertile men having elevated levels of free radicals in sperm [1]. Therefore, a comprehensive assessment of male reproductive capacity should include an assessment of sperm oxidative stress. Human semen contains several antioxidant systems to remove oxygen free radicals and prevent oxidative tissue damage. They are classified as enzymatic and non-enzymatic antioxidants [2]. The formation of the enzymatic antioxidant system, the elements of which vary between organisms, is a crucial breakthrough in spermatogenesis to ensure the protection of sperm against oxidative stress [3]. Under normal conditions, reactive oxygen species (ROS) are neutralized by enzymes such as peroxide dismutase, catalase, glutathione peroxidase, and glutathione transferase [4].

Glutathione S-transferases (GSTs) comprise a superfamily of ubiquitously expressed multifunctional enzymes that play a pivotal role in protecting cells against oxidative stress. Among them, the *GSTM1* gene, encoding the glutathione S-transferase Mu-1, is mutated and is devoid of any specific enzymatic activity (*GSTM1*-null genotype). Interestingly, half of the Caucasian population carry the null genotype. Therefore, *GSTM1* gene deletion might be correlated with an increased susceptibility to diseases associated with oxidative stress. There are also studies reporting that *GSTM1* might be a critical isozyme in the detoxification of oxidative stress products [2,5,6]. 

An association has been demonstrated between *GSTM1* polymorphism, markers of oxidative stress, and damage in spermatozoa and seminal plasma in subjects with idiopathic male infertility. Infertile individuals with the *GSTM1*-null genotype are more susceptible to oxidative stress than *GSTM1-positive* infertile males. Furthermore, the *GSTM1-null* genotype has been associated with higher ROS, protein carbonyl, and malondialdehyde (MDA) levels [2].

The aim of the present study is to investigate the effect of *GSTM1* polymorphism and static redox potential (sORP) to the basic seminal parameters of fertile and infertile men. Furthermore, our study’s aim is to examine whether testing for *GSTM1* polymorphism could be a potential biomarker in male infertility investigation.

## 2. Materials and Methods

### 2.1. Sample Collection

The case–control study was conducted at “Alexandra” University Hospital in collaboration with the sperm cryopreservation bank “Cryogonia” and a written informed consent was obtained from all the involved patients. The study included 90 Caucasian males divided into two groups, namely the study group and the control group. The study group consisted of semen samples from 51 men identified as infertile according to the WHO 2010 guidelines (study group). Control group consisted of 39 fertile men with normal seminal parameters according to the WHO 2010 guidelines, and at least one successful pregnancy with their partner without assisted reproductive technologies. Exclusion criteria for both groups include evidence of any other fertility-related disease, such as prostate cancer, cryptorchidism, varicocele, diabetes, seminal infections, or karyotype abnormalities. Moreover, individuals with obesity (body mass index greater than 30 kg/m^2^), systematic alcohol consumption, or active nicotine abuse were also excluded. Each subject donated 1 mL (patient cohort) or 0.5 mL (donor cohort) of ejaculated semen obtained by masturbation after a minimum of 4 days of abstinence.

### 2.2. Semen Analysis

Each sample was subjected to conventional semen analysis according to the recommendations, semen evaluation protocols, and standards of the World Health Organization (WHO). More specifically, seminal volume, sperm concentration, motility, and morphology were analyzed 30 min after liquefaction.

### 2.3. DNA Extraction and Detection of GSTM1 Polymorphism

DNA was isolated from all sperm samples using a commercial PureLink™ Genomic DNA Mini Kit (Thermo Fisher Scientific, Waltham, MA, USA). DNA quantification was performed via spectrophotometer, and DNA integrity was verified by agarose electrophoresis. Polymerase Chain Reaction was performed to detect *GSTM1* polymorphism. A Taq DNA polymerase kit (New England Biolabs, Ipswich, MA USA) was used, and the primers were the following: GSTM1F 5′-GAACTCCCTGAAAAGCTAAAGC-3′ and GSTM1R 5′-GTTGGGCTCAAATATACGGTGG-3′ [7]. The conditions of the PCR were as follows: 94 °C for 10 min, 94 °C for 1 min, 58 °C for 1 min, 72 °C for 1 min. This was repeated for 35 cycles, with a final elongation step at 72 °C for 10 min. Subsequently, the PCR products were electrophoresed on 2% agarose gel and visualized under UV light. A single band at 219 bp corresponded to the presence of the *GSTM1*.

### 2.4. Evaluation of Oxidative Stress Using the MiOXSYS^TM^ System

The MiOXSYS^TM^ system (GryNumber Health Group, Vilnius, Lithuania) provides an evaluation of the static redox potential (sORP), which represents an integrated measure of the balance between total levels of oxidants and antioxidants in a biological system or sample, such as human semen. The MiOXSYS^TM^ system requires a small semen volume (30 μL) and produces results in less than 5 minutes. The measurement of sORP offers the andrology and embryology laboratory an efficient tool for the measurement of oxidative stress. The assay provides the possibility to directly assess the oxidative potential in semen samples in contrast to oxidative stress markers such as ROS, TAC or MDA. Its advantages compared to other methods of oxidative stress evaluation include short duration, technical simplicity, as well as small sample volume required for the measurement.

### 2.5. Statistical Analysis

The χ^2^-test was performed to compare GSTM1-null genotype frequencies between groups. Odds ratios (ORs) and 95% confidence intervals (CIs) were calculated to evaluate the association between variant genotype and group. *T*-test for two independent samples (or Mann–Whitney U, if this was deemed necessary) was performed to compare variant genotype and conventional semen parameters by group. Pearson’s correlation coefficient was used to determine the effect of sORP in semen parameters. The data were analyzed using the Statistical Package for Social Sciences software version 20.0 (SPSS Inc., Chicago, IL, USA), and *p* < 0.05 was considered statistically significant.

## 3. Results

### 3.1. General Characteristics and Conventional Semen Parameters in Study and Control Groups

The present case–control study included 90 Caucasian males, of whom 51 (56.67%) and 39 (43.33%) were included in the study and control groups, respectively. The mean age of the infertile and fertile individuals was 38.67 ± 6.46 years and 39.21 ± 6.64 years, respectively. Both groups were age matched, and there was no significant difference in the age distribution between the control and study group. There was no difference in BMI and lifestyle factors between both groups.

Conventional semen parameters, including sperm motility, concentration, and morphology for both groups are reported in Table 1. Sperm concentration was lower in the study group compared to controls, a finding with statistical significance (26.78 × 10^6^/mL vs. 90.92 × 10^6^/mL, *p* < 0.001). Likewise, total sperm count was statistically significantly restricted in infertile men compared to in those who were fertile (81.49 × 10^6^ vs. 275.89 × 10^6^, *p* < 0.001). Moreover, infertile subjects presented decreased total sperm motility compared to the controls, an observation that reached statistical significance (47.80% vs. 55.62%, *p* < 0.001). Accordingly, the immotile sperm percentage was statistically significantly higher in the study group than in the controls (48.86% vs. 21.56%, *p* < 0.001). In addition, sperm morphology was found to be diminished in infertile men compared to fertile ones (2.82% vs. 8.41%, *p* < 0.001).

### 3.2. GSTM1-Null Genotype Frequencies and Association with Conventional Semen Parameters

The genotype distribution of *GSTM1* polymorphism in patients with infertility and in the controls is presented in Table 2. The frequency of the *GSTM1*-null genotype was higher in infertile male individuals (31/51; 60.78%) than in the controls (16/39; 41.03%), with an OR of 2.228, but it was not statistically significant. Furthermore, the presence or absence of *GSTM1* polymorphism was studied in relation to sperm concentration, motility, and morphology parameters. This analysis aimed to investigate whether the absence of *GSTM1* gene affects conventional semen parameters, such as morphology and motility, in fertile and infertile men. For the control group (Table 3), the presence of the *GSTM1*-null genotype was associated with a higher pH (7.600 ± 0.110 vs. 7.400 ± 0.100, *p* = 0.033), lower percentage of slow progressive motility (39.94 ± 9.81 vs. 45.91 ± 9.01, *p* = 0.043) and lower percentage of morphologically typical spermatozoa (7.19 ± 2.83 vs. 9.26 ± 2.94, *p* = 0.020). Moreover, the percentage of immotile spermatozoa was higher in *GSTM1*-null genotype carriers (26.31 ± 13.45 vs. 18.26 ± 7.62, *p* = 0.053), but it was not statistically significant. Interestingly, there were no statistically significant differences between the *GSTM1*-null genotype and conventional semen parameters in infertile male individuals (Table 4).

### 3.3. Association of Static Oxidation-Reduction Potential (sORP) with Conventional Semen Parameters

**Figure 1:**
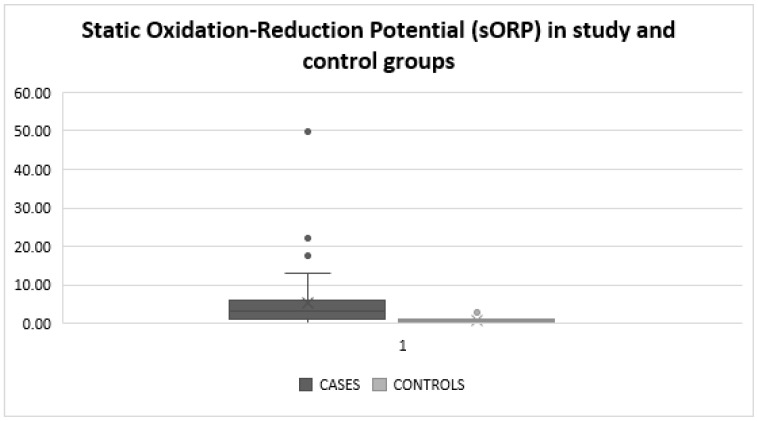
Boxplot presenting Static Oxidation-reduction Potential (sORP) data in study and control groups.

Τhe measurement of oxidative stress using the MiOXSYS™ system was performed on 52 individuals in total, with an average value of 4.58 ± 7.72 mV. More specifically, in the control group, the measurement of oxidative stress was performed in nine individuals with an average value of 0.91 ± 0.8 mV, while in the infertile group, the measurement of oxidative stress was performed in 43 individuals with an average value of 5.35 ± 8.29 mV. The two groups differed significantly (*p* = 0.0013), indicating that an excess of ROS was detected in infertile men compared to fertile ones. Figure 1 presents the averages and distribution of Static Oxidation-reduction Potential data in fertile and infertile men.

Regarding the association of oxidation-reduction potential with conventional semen parameters in infertile individuals (Table 5), sperm concentration and total sperm count had a statistically significant negative correlation with increased sORP values. Likewise, slow progressive motility was negatively correlated with oxidation-reduction potential. On the other hand, in the control group (Table 6), there was a negative correlation between the increase in sORP values and total motility percentage. Additionally, there was a positive correlation between increased sORP and immotile spermatozoa. All the above-mentioned correlations indicate that when seminal plasma sORP increases, semen count and motility parameters decline in infertile men and only motility parameters decline in fertile men.

Interestingly, a negative correlation was found when analyzing the relationship between sORP values in the study group and viscosity of the seminal fluid (Table 7). 

## 4. Discussion

Genetic variants in *GST* genes may lead to detoxification system imbalance, thereby increasing susceptibility to oxidative stress damage and increased risk for male infertility [8,9]. In particular, several epidemiological studies have shown that the *GSTM1*-null genotype, which results in total enzyme deficiency, is associated with increased susceptibility to oxidative-stress-related diseases. The possible relationship of *GSTM1* gene deficiency with male infertility has been studied extensively, but the results of the studies vary between different populations [10].

The present case–control study focused on the potential impact of the *GSTM1* polymorphism and redox potential on the risk for idiopathic male infertility. Our results demonstrated that the *GSTM1*-null genotype was present at a higher frequency in infertile men than in the fertile control group, with a 2.22-fold increase for the risk of male infertility. Thus, an increased risk of the *GSTM1* polymorphism for developing male factor infertility is supported. 

Our results are in agreement with several studies that reveal *GSTM1*-null genotype as a potential risk factor for male idiopathic infertility by affecting semen quality [11,12,13,14,15,16]. Numerous population studies have suggested a negative effect of the *GSTM1*-null genotype on male infertility, with patients carrying the *GSTM1*-null genotype having a lower sperm concentration and sperm count. Fertile males with the *GSTM1*-null genotype had a lower sperm concentration but normal sperm count [17,18]. More importantly, a similar relative risk for male factor infertility was observed in patients with the *GSTM1*-null genotype. The same study showed that the combination of deletion genotypes of GST genes pose an even higher risk for infertility [19].

Impaired semen quality is reported in infertile men regarding sperm concentration, count, motility, and morphology compared to fertile ones. In our study, fertile individuals carrying the *GSTM1*-null genotype were found to have a lower percentage of typical spermatozoa regarding morphology and lower slow progressive motility, highlighting the negative effect of the presence of *GSTM1* polymorphism in conventional semen parameters in fertile males. Notably, Aydemir et al. showed that lower sperm concentrations and higher levels of oxidative stress and damage markers are presented in infertile males with the *GSTM1*-null genotype compared to those with the *GSTM1*-positive genotype. However, no significant difference in genotype distribution for the *GSTM1* variant between idiopathic infertile subjects and fertile ones was observed [2]. Another study also demonstrated lower sperm concentration and motility in infertile men carrying the *GSTM1*-null genotype compared to fertile ones with the *GSTM1*-positive genotype. Similarly, the frequency of the *GSTM1*-null genotype was significantly higher in infertile individuals than in fertile ones. Their findings are consistent with our research results [20].

Moreover, an increased risk for male infertility of *GSTM1*-null genotype was revealed in a previous meta-analysis. Interestingly, an even higher risk was reported upon a subgroup analysis of Caucasians [21]. Similarly, a subsequent meta-analysis concluded that the *GSTM1*-null polymorphism contributes to a significant increased risk for male infertility. Therein, significant associations were also observed in subgroups of Caucasian populations but not in Asian ones [22].

On the contrary, in a meta-analysis conducted by Economopoulos et al., the *GSTM1*-null genotype was not statistically associated with male infertility, underscoring the need for the accumulation of data regarding variants of GST genes [23]. However, in a recent study, researchers proposed the *GSTM1*-null genotype as a potential genetic risk factor for male infertility, interfering with certain oxidative stress markers (i.e., total antioxidant capacity and nitric oxide) in infertile men [24].

With regard to oxidative stress variations, our results demonstrated an excess of oxidation-reduction potential in infertile men compared to fertile ones. Notably, poor semen quality, including low sperm concentration and count, higher percentage of slow progressive motility and immature forms, was reported upon redox potential elevation. Surprisingly, increases in sOPR had a negative impact on the semen motility characteristics of fertile males.

The measure of sORP is considered to be a better indicator of semen quality, providing reliable results for oxidative stress. Agarwal et al. standardized the sORP test in semen using the MiOXSYS System and reported that higher sORP levels were associated with poor sperm parameters, deteriorating the fertility status of subjects. Negative correlations emerged for conventional semen parameters, including concentration, total sperm count, motility, and morphology, indicating that oxidative stress impairs these parameters. The authors proposed testing sORP as an objective and accurate method, which in conjunction to routine semen analysis can reliably differentiate fertile from infertile men [25,26]. However, certain limitations should be taken into consideration regarding the difficulty to assess highly viscous semen using the MiOXSYS system, or conventional semen parameters, such as sperm morphology. 

## 5. Conclusions

In conclusion, our study showed that the *GSTM1*-null genotype does not significantly affect the semen parameters of the infertile group. On the other hand, the existence of the *GSTM1*-null genotype in the control group was associated with lower slow progressive motility and less typical spermatozoa. Regarding sORP values, we found a negative correlation with total count, concentration, and slow progressive motility in the infertile group. In the fertile control group, there was a positive correlation with immotility percentage and a negative correlation in total motility. Ultimately, the importance of our results lies in the confirmation that the *GSTM1*-null genotype and sORP values affect both fertile and infertile males in different ways. Further studies are needed to evaluate the combined or independent use of *GSTM1* genotyping as a potential biomarker for male infertility assessment. In addition, redox potential is considered to be a useful indicator of semen quality, distinguishing infertile from fertile males by their sORP values. Accordingly, genetic testing along with an oxidative stress test may contribute to the identification of those infertile patients with increased risk for abnormal semen parameters.

## Figures and Tables

**Table 1:** Comparison of conventional semen parameters in control (*n* = 39) and study (*n* = 51) groups.

Semen Parameters	N_0_	Control Group	N_1_	Study Group	*p*-Value
pH	9	7.53 ± 0.14	45	7.62 ± 0.21	0.240
Days of Abstinence	39	4.10 ± 1.33	51	3.88 ± 2.20	0.585
Semen Volume (mL)	39	2.94 ± 1.16	51	3.07 ± 1.77	0.386
Sperm Concentration (10^6^/mL)	39	90.92 ± 50.40	51	26.78 ± 29.04	0.000
Total Sperm Count (10^6^)	39	275.89 ± 208.42	51	81.49 ± 101.35	0.000
Total Motility (%)	39	55.62 ± 6.50	51	47.80 ± 12.25	0.000
Rapid Progressive Motility (%)	39	2.15 ± 5.22	51	7.35 ± 9.08	0.001
Slow Progressive Motility (%)	39	43.46 ± 9.69	51	20.71 ± 8.18	0.000
Non-progressive Motility (%)	39	32.67 ± 9.46	51	22.88 ± 14.24	0.000
Immotility (%)	39	21.56 ± 11.00	51	48.86 ± 14.67	0.000
Typical Morphology (%)	39	8.41 ± 3.04	51	2.82 ± 2.33	0.000
Head Abnormalities (%)	1	94	21	98.52 ± 0.75	0.000
Midpiece Abnormalities (%)	1	18	21	28.52 ± 7.80	0.202
Tail Abnormalities (%)	1	10	21	14.57 ± 9.85	0.655
Immature Forms (%)	1	0	21	6.67 ± 3.28	0.061
Viscosity (cps)					
normal		5 (12.8%)		12 (23.5%)	
abnormal		34 (87.2%)		39 (76.5%)	0.198
Liquefaction					
normal		3 (7.7%)		2 (3.9%)	
abnormal		36 (92.3%)		49 (96.1%)	0.439

Values are mean ± SD or number (column percentage). *t*-test independent sample or Chi-square test was used.

**Table 2:** Frequencies of *GSTM1*-null genotype in control (*n* = 39) and study (*n* = 51) groups.

		Group		Risk Estimate		
		Control	Study	OR	95% CI for OR	*p*-Value
GSTM1	no	23 (59.0%)	20 (39.2%)	2.228	[0.952 5.215]	0.063
	yes	16 (41.0%)	31 (60.8%)			

Pearson’s Chi-square test was used.

**Table 3:** Association of *GSTM1*-null genotype with conventional semen parameters (control group).

Semen Parameters	N_0_	GSTM1 (No)	N_1_	GSTM1 (Yes)	*p*-Value
pH	3	7.400 ± 0.100	6	7.600 ± 0.110	0.033
Days of Abstinence	23	4.304 ± 1.636	16	3.813 ± 0.655	0.420
Semen Volume (mL)	23	2.743 ± 1.158	16	5.206 ± 8.027	0.138
Sperm Concentration (10^6^/mL)	23	95.39 ± 52.63	16	84.50 ± 47.92	0.568
Total Sperm Count (10^6^)	23	265.70 ± 202.13	16	290.54 ± 223.02	0.808
Total Motility (%)	23	55.65 ± 6.44	16	55.56 ± 6.81	0.976
Rapid Progressive Motility (%)	23	1.13 ± 2.99	16	3.63 ± 7.21	0.113
Slow Progressive Motility (%)	23	45.91 ± 9.01	16	39.94 ± 9.81	0.043
Non-progressive Motility (%)	23	34.70 ± 7.25	16	29.75 ± 11.59	0.244
Immotility (%)	23	18.26 ± 7.62	16	26.31 ± 13.45	0.053
Typical Morphology (%)	23	9.26 ± 2.94	16	7.19 ± 2.83	0.020
Head Abnormalities (%)	0		1	94.00±	
Midpiece Abnormalities (%)	0		1	18.00±	
Tail Abnormalities (%)	0		1	10.00±	
Immature Forms (%)	0		1	0.000±	
sORP (mV/10^6^ sperm/mL)	3	0.690 ± 0.345	6	1.023 ± 0.959	0.796
Viscosity (cps)					
normal		3 (13.0%)		2 (12.5%)	
abnormal		20 (87.0%)		14 (87.5%)	0.960
Liquefaction					
normal		2 (8.7%)		1 (6.2%)	
abnormal		21 (91.3%)		15 (93.8%)	0.778

Values are mean ± SD or number (column percentage). Mann–Whitney U test or Chi-square test was used.

**Table 4:** Association of *GSTM1*-null genotype with conventional semen parameters (study group).

Semen Parameters	N_0_	GSTM1 (No)	N_1_	GSTM1 (Yes)	*p*-Value
pH	19	7.621 ± 0.190	26	7.623 ± 0.234	0.972
Days of Abstinence	20	3.750 ± 2.770	31	3.968 ± 1.779	0.268
Semen Volume (mL)	20	2.580 ± 1.240	31	3.381 ± 1.992	0.202
Sperm Concentration (10^6^/mL)	20	24.96 ± 25.36	31	27.95 ± 31.54	0.847
Total Sperm Count (10^6^)	20	57.87 ± 59.78	31	96.72 ± 119.35	0.657
Total Motility (%)	20	47.75 ± 11.26	31	47.84 ± 13.04	0.787
Rapid Progressive Motility (%)	20	6.50 ± 7.26	31	7.90 ± 10.16	0.627
Slow Progressive Motility (%)	20	20.40 ± 7.05	31	20.90 ± 8.93	0.764
Non-progressive Motility (%)	20	21.85 ± 11.73	31	23.55 ± 15.80	0.802
Immotility (%)	20	51.25 ± 12.31	31	47.32 ± 16.01	0.429
Typical Morphology (%)	20	2.20 ± 1.11	31	3.23 ± 2.80	0.456
Head Abnormalities (%)	9	98.222 ± 0.833	12	98.750 ± 0.622	0.081
Midpiece Abnormalities (%)	9	26.444 ± 6.126	12	30.083 ± 8.785	0.200
Tail Abnormalities (%)	9	15.222 ± 11.032	12	14.083 ± 9.337	0.475
Immature Forms (%)	9	6.667 ± 2.828	12	6.667 ± 3.701	0.721
sORP (mV/10^6^ sperm/mL)	17	6.140 ± 11.481	26	4.827 ± 5.521	0.737
Viscosity (cps)					
normal		5 (25.0%)		7 (22.6%)	
abnormal		15 (75.0%)		24 (77.4%)	0.842
Liquefaction					
normal		0 (0.0%)		2 (6.5%)	
abnormal		20 (100%)		29 (93.5%)	0.247

Values are mean ± SD or number (column percentage). Mann–Whitney U test or Chi-square test was used.

**Table 5:** Association of Static Oxidation-reduction Potential (sORP) with conventional semen parameters (study group).

Semen Parameters	*N*	Pearson’s Correlation	*p*-Value
pH	43	−0.190	0.222
Days of Abstinence	43	−0.060	0.703
Semen Volume (mL)	43	0.226	0.145
Sperm Concentration (10^6^/mL)	43	−0.369	0.015
Total Sperm Count (10^6^)	43	−0.315	0.040
Total Motility (%)	43	−0.278	0.071
Rapid Progressive Motility (%)	43	−0.280	0.069
Slow Progressive Motility (%)	43	−0.348	0.022
Non-progressive Motility (%)	43	0.277	0.072
Immotility (%)	43	0.275	0.075
Typical Morphology (%)	43	−0.051	0.743
Head Abnormalities (%)	21	0.189	0.411
Midpiece Abnormalities (%)	21	−0.059	0.801
Tail Abnormalities (%)	21	0.006	0.980
Immature Forms (%)	21	0.421	0.057

Pearson correlation was used.

**Table 6:** Association of Static Oxidation-reduction Potential (sORP) with conventional semen parameters (control group).

Semen Parameters	*N*	Pearson’s Correlation	*p*-Value
pH	9	0.259	0.500
Days of Abstinence	9	−0.044	0.910
Semen Volume (mL)	9	0.274	0.476
Sperm Concentration (10^6^/mL)	9	−0.375	0.320
Total Sperm Count (10^6^)	9	−0.074	0.851
Total Motility (%)	9	−0.701	0.035
Rapid Progressive Motility (%)	9	−0.021	0.957
Slow Progressive Motility (%)	9	−0.473	0.198
Non-Progressive Motility (%)	9	−0.554	0.122
Immotility (%)	9	0.701	0.035
Typical Morphology (%)	9	0.028	0.943
Head Abnormalities (%)	1		
Midpiece Abnormalities (%)	1		
Tail Abnormalities (%)	1		
Immature Forms (%)	1		

Pearson correlation was used.

**Table 7:** Association of Static Oxidation-reduction Potential (sORP) with Viscosity and Liquefaction in both groups.

**Control Group**					
	**N1**	**Normal**	**N2**	**Abnormal**	***p*-Value**
Viscosity (cps)	2	1.925 ± 1.266	7	0.623 ± 0.370	0.079
Liquefaction	0		9	0.912 ± 0.795	
**Study Group**					
	**N1**	**Normal**	**N2**	**Abnormal**	***p*-Value**
Viscosity (cps)	9	11.767 ± 15.09	34	3.645 ± 4.252	0.022
Liquefaction	0		43	5.345 ± 8.293	

Mann–Whitney U test was used.

## Data Availability

Not applicable.
